# Auditory Event-Related “Global Effect” Predicts Recovery of Overt Consciousness

**DOI:** 10.3389/fneur.2020.588233

**Published:** 2021-01-08

**Authors:** Pauline Perez, Mélanie Valente, Bertrand Hermann, Jacobo Sitt, Frédéric Faugeras, Sophie Demeret, Benjamin Rohaut, Lionel Naccache

**Affiliations:** ^1^PICNIC Lab Team, INSERM, U 1127, CNRS UMR 7225, Faculté de Médecine de Sorbonne Université, UMR S 1127, Institut du Cerveau et de la Moelle épinière, ICM, Hôpital Pitié-Salpêtrière, Paris, France; ^2^Assistance Publique Hôpitaux de Paris (APHP), Hôpital Pitié-Salpêtrière, Department of Clinical Neurophysiology, Paris, France; ^3^Assistance Publique Hôpitaux de Paris (APHP), Hôpital Henri-Mondor, Department of Neurology, Créteil, France; ^4^Assistance Publique Hôpitaux de Paris (APHP), Hôpital Pitié-Salpêtrière, Department of Neurology, Paris, France; ^5^Faculté de Médecine Pitié-Salpêtrière, Sorbonne Université, Paris, France

**Keywords:** disorder of consciousness (DOC), prognosis, EEG–electroencephalogram, critical care, evoked potential

## Abstract

**Objective:** To explore whether the presence of an event-related potential (ERP) “global effect” (GE+)—that corresponds to a correlate of conscious processing in the local–global auditory task—predicts behaviorally overt consciousness recovery in a large cohort of patients suffering from disorders of consciousness (DOC).

**Methods:** We conducted a prospective study on all DOC patients evaluated during the 2009–2018 period. Behavioral examination included Coma Recovery Scale-Revised (CRS-R) scores and bedside high-density EEG recordings. Consciousness recovery was evaluated at 6 months by a structured phone interview. The predictive value of a GE+ was calculated both on survivors and on all patients.

**Results:** A total of 236 patients with a documented outcome and technically valid EEG recordings could be included. Among them, 66 patients had a GE+ status (28%). Presence of GE+ predicted behaviorally overt consciousness recovery in survivors with high specificity (Sp = 84%) and high positive predictive value (PPV = 80%) but with low sensitivity (Se = 35%) and low negative predictive value (NPV = 42%). Positive likelihood ratio (LR+) of GE+ was superior to LR+ of initial clinical status and of ERP effect indexing unconscious auditory processing [local effect (LE)].

**Interpretation:** Our results demonstrate that the presence of a bedside ERP GE+ is highly predictive of behaviorally overt consciousness recovery in DOC patients, regardless of the delay, of behavioral status, and of the etiology of brain dysfunction. However, the absence of this effect is not a reliable predictor of negative outcome. This study provides Class III evidence that the presence of an ERP “global effect” predicts consciousness recovery in DOC patients.

## Introduction

The ability to predict consciousness recovery in patients suffering from disorders of consciousness constitutes a major clinical and neuroscientific challenge. A precise behavioral diagnosis of the type of disorder of consciousness (DOC) constitutes a first specific predictor of consciousness recovery, in addition to etiology and age. Indeed, recent studies converged on demonstrating that an early distinction between a minimally conscious state (MCS) and a vegetative state (VS, also coined unresponsive wakefulness syndrome or UWS) predicts consciousness recovery (1–4). However, this distinction requires a genuine behavioral expertise, as evidenced both by a rate of ~40% of initial diagnostic errors before expert behavioral evaluation (5) and by frequent errors in the use of the revised version of the Coma Recovery Scale (CRS-R), which is the gold standard tool to assess consciousness in DOC patients (6). Moreover, recordings of brain activity with PET-glucose imaging, functional MRI (fMRI), or EEG during cognitively passive or active conditions invariably revealed that around 15% of DOC patients diagnosed by CRS-R experts as being in a non-conscious VS/UWS show neural evidence in favor of an MCS or of a fully conscious state (7–16). All these limitations call for the use of additional diagnostic specific markers derived from brain activity that may also be useful to predict consciousness recovery.

In this context, we aimed at exploring the prognosis value of such a brain activity diagnostic marker that we previously conceived as a specific signature of conscious access to the violation of auditory regularities: the event-related EEG global effect (GE) (17). More precisely, we designed an auditory paradigm to probe cerebral responses to violations of temporal regularities that are either local in time (intra-trial) or global across several seconds and several trials. Local regularity violations (local effect or LE) led to an early response in the auditory cortex, independent of attention or the presence of a concurrent visual task, whereas global violations led to a late and spatially distributed response. Interestingly, GE was found significant in each of the healthy controls who attended to the series and sounds and counted occurrences of global violations. However, in the absence of instructions, this effect was present exclusively in those subjects who could report the existence of violations of global regularities. We could detect the GE in individual subjects using fMRI and both scalp and intracerebral event-related potentials (ERPs) (18), and more recently with pupillometry (19). Applied to DOC patients, our initial logic was to infer that the presence of such a signature of conscious access to a specific perceptual attribute (violations of global regularities) would, by definition, require the patient to be in a conscious state. In other terms, by probing conscious access to violations of the global regularity, we would indirectly probe conscious states that are prerequisite to enable conscious access. In a set of six studies (11, 12, 17, 20–22), we confirmed that GE was mostly present in clinically conscious patients, as compared to MCS and to VS/UWS patients.

Encouraged by this cumulative set of results, we hypothesized that GE could be a specific tool to probe conscious patients among non-communicating patients who do not show univocal behavioral overt evidence of consciousness. More precisely, we aimed at testing if GE could be in advance, relative to behavior, to detect consciousness in these patients. We were encouraged in this hypothesis by a previous report in which the only two, out of 20, behaviorally VS/UWS patients with a GE improved to MCS, respectively, 3 and 4 days after ERP recording (20), strongly suggesting that they were actually conscious when tested.

In this study, we confirmed our hypothesis on a large cohort of patients with a documented outcome.

## Methods

### Population

Patients hospitalized in the neuro intensive care unit of the Pitié-Salpêtrière Hospital for a multimodal assessment of consciousness during the 2008–2019 period were included in the present study. During their stay, several evaluations and exams were performed including neurological clinical assessment and CRS-R scoring, structural brain MRI, high-density EEG, ERP with the “local–global” auditory task. This “routine care research” study was approved by the CPP IDF1 ethics committee.

### Standard Protocol Approvals, Registrations, and Patient Consents

This research was approved by the local ethics committee Comite de Protection des Personnes Ile de France 1 (Paris, France) under the code “Recherche en soins courants” (NEURODOC protocol, n° 2013-A01385-40). Patient's family gave their informed consent for the participation of their relative, and all investigations conformed to the Declaration of Helsinki and the French regulations.

### Coma Recovery Scale-Revised Scoring and Outcome Evaluation

The state of consciousness was determined by neurologists or intensivists who are trained users of the French version of CRS-R (LN, FF, BR, BH, PP). We used the CRS-R score measured immediately before “local–global” task ERP recording. The primary outcome of this study was patient conscious status at 6 months and was collected through a structured phone interview with patient's relatives (Supplementary Table 1). This structured interview was inspired by the CRS-R items and aimed at distinguishing conscious individuals on the one hand (i.e., univocal functional communication) from VS/UWS and MCS individuals on the other hand. Subsequent analyses were conducted separately on the whole population of patients (including deaths) and on survivors only.

### Local–Global Event-Related Potential Paradigm

The local–global paradigm derives from the classic auditory oddball, and it has been reported in several articles since its original publication in 2009 (17). This paradigm crosses two types of auditory regularities and enables to test neural responses to their respective violations. Each trial contains five sounds. While the first four are always identical, the fifth can be identical or different (local regularity). These trials are included in blocks structured by an inter-trial regularity (global regularity): in half of the blocks, the global regularity fits with the local regularity (more frequent trials have five identical sounds), whereas in the other half of blocks, global and local regularities are in opposition (more frequent trials have a distinct fifth sound). Habituation trials were not used to compute local effect (LE) and GE analyses. By comparing neural responses to local deviant vs. local standard trials, one can compute the LE, while the global deviant vs. global standard comparison defined the GE. See Supplementary Methods for more details.

### EEG Preprocessing

ERPs were recorded at 250 Hz with a 256-electrode geodesic sensor net (EGI®, Oregon, USA) referenced to the vertex. Trials were band-pass filtered (0.5–45 Hz), then segmented in epochs ranging from −200 ms to +1,344 ms from first sound onset. Electrodes with voltages exceeding 100 μV in more than 50% of the epochs were removed. Moreover, voltage variance was computed across all correct electrodes. Electrodes with a voltage variance Z-score higher than 4 were removed. This process was repeated four times. Bad electrodes were interpolated using a spline method (23). Epochs were labeled as bad and discarded when voltage exceeded 100 μV in more than 10% of electrodes. Moreover, voltage variance was computed across all correct epochs, and epochs with a Z-score larger than 4 were removed. This process was also repeated four times. Remaining stimulus-locked epochs were averaged and digitally transformed to an average reference. An 800s baseline correction (before fifth sound onset) was applied. EEG recordings had to satisfy the following two criteria to be further analyzed: they should include a minimum of 75% valid channels and 30% of valid epochs. Preprocessing was implemented using MNE-Python environment.

### Event-Related Potential Analyses and Statistics

For individual subject statistics, unpaired Welch's tests were performed for each time sample. An effect was considered significant if it satisfied the following triple-threshold criterion that we previously used and validated (17): *p* < 0.05 on a minimum of five consecutive samples (20 ms) and on a minimum of 10 contiguous electrodes during the expected time window of the corresponding ERP effect. For the LE, an ERP was expected from 100 ms after first sound onset to the end of epoch for the LE, whereas the time window ranged from 200 ms to end of epoch for the GE. This 200-ms criterion was determined on the basis of previous results and on the following considerations: (i) while early and late mismatch negativity Mismatch Negativity (MMN) components occur within a 100-m−200-ms temporal window (24), the onset of GE typically occurs around 250 ms at the group-level statistics with a slope beginning a few tens of milliseconds earlier [see, for instance, Bekinschtein et al. (17) and Faugeras et al. (21)]; (ii) at the single-subject statistics, significant GE can occur around 200 ms [see, for instance, Figure 4 in Faugeras et al. (21)]; (iii) note also that the local–global task design was conceived to cancel LE components when computing GE by balancing local standard and local deviant trials across global standard and global deviant trials (17). We therefore adopted the 200 ms criterion for GE onset.

Moreover, *p*-value of any cluster of interest satisfying this triple-threshold criterion also had to be smaller, or identical but longer in time, than any other cluster occurring before the relevant time window. This algorithmic procedure was implemented and batched in Python language. So, we did not impose any constraint of topography or polarity of ERP effects for both LE and GE aspects.

### Statistics

The statistical analysis was performed with R software for the frequentist tests and with JASP 0.10.2.0 software for Bayesian tests.

More specifically, we used frequentist approach to compute sensitivity (Se), specificity (Sp), positive predictive value (PPV), negative predictive value (NPV), and positive (LR+) and negative (LR-) likelihood ratio. We also calculated Bayes factor (BF) of the corresponding contingency tables (25) using the JASP software (26) in order to test the following hypothesis: GE+ group > GE- group. We report the BFs using the Raftery terminology (27).

### Data Availability

Data are available upon reasonable request but cannot be made open due to ethics protocol requirement and the sensitive nature of patient's data.

## Results

During the 2009–2018 period, we recorded high-density ERPs during the active “local–global” task in 429 non-communicating patients addressed to our team at the Pitié-Salpêtrière University Hospital in Paris for a diagnostic and prognostic evaluation of consciousness.

**Figure 1:**
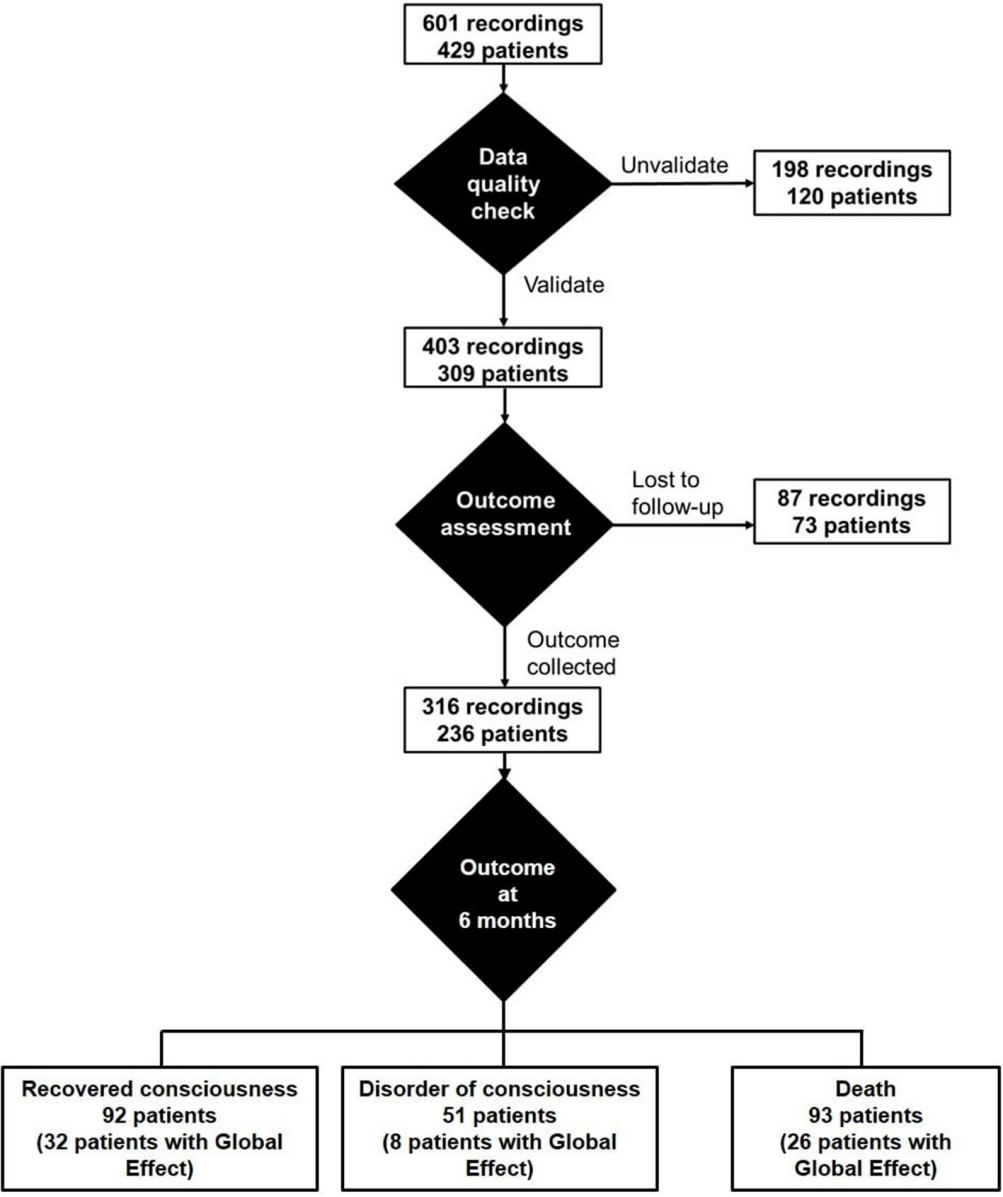
Flowchart.

Some patients were recorded several times (for a total of 601 recordings performed on 429 distinct patients, see flowchart in Figure 1). Only 403 recordings (67%) satisfied data quality criterion defined in previous studies (see Methods). These 403 valid recordings corresponded to 309 different patients. We used the following two criteria to select only one of these multiple recordings: (i) in order to maximize the number of recordings showing a GE (GE+), if a GE was significant on one or several recordings, we kept the first recording showing a GE; and (ii) when no GE was significant on any of the recordings (GE-), we kept the first recording. Behavioral labeling of these patients was based on the best CRS-R scores (from a number of individual scorings ranging from 2 to 5) and revealed that while most of them were either in MCS (*N* = 141) or in VS/UWS (*N* = 138), some of them were in a conscious exit-MCS (EMCS) state (*N* = 30). We kept all these patients in the main analyses of our results (see below for further analyses) because each of them was referred to us by their clinicians who could not determine their state of consciousness. Among these 309 recordings from distinct patients, 80 were GE+ (25.8%). Both the “patients-based” (*N* = 309) and the “recordings-based” (*N* = 403) analyses revealed the same pattern of results. For the sake of concision, we report in the main text the patients-based analyses (see Supplementary Tables 2, 3 for the detailed results of “recordings-based” results).

### Description of Patients

This 309-patient cohort was mainly composed of men (66.3% men; 47.6% younger than 45 years old; Supplementary Table 6). The most common etiology was anoxia (32.7%), then traumatic brain injury (TBI) (22.7%). The other main etiologies included stroke or hematoma as well as encephalitis or toxic encephalopathy (see Supplementary Table 6 for details). We regrouped all of these patients under the label “Other.” The delay between the brain lesion and the evaluation was <3 months for 219 patients (70.9%).

GE+ was more frequent in EMCS (15/29 = 51.7%) than in MCS (33/142 = 23.2%) and in VS/UWS (32/138 = 23.2%) patients (Fisher exact test, *p* = 0.007). No difference of GE+ proportion was found between MCS and VS patients [Fisher exact test, *p* = 1; BF_+0_ = 0.16] and between MCS+ with MCS- patients (27.8 vs. 18.6%, respectively; Fisher exact test, *p* = 0.23; BF_+0_ = 1.07). Note the absence of effect of etiology on GE+ proportion both on the whole cohort (*N* = 309; Fisher exact test, *p* = 0.13) and on the subgroup of patients with a documented outcome (*N* = 236; *p* = 0.25).

### Outcome and Consciousness Recovery

Among these 309 patients, we could document the outcome at 6 months in 236 patients (76%) including 92 (39%) who recovered consciousness, 51 (22%) who did not, and 93 (39%) who died. Among the missing data (*N* = 73), the initial conscious status was VS/UWS for 36 patients, MCS for 29 patients (MCS- = 16; MCS+ = 13), and EMCS for eight patients. The proportion of VS (UWS)/MCS/EMCS patients did not differ between the group of missing data and the group with a documented outcome (Fisher exact test, *p* = 0.9, BF_+0_ = 0.53).

We first replicated the relevance of following three classical predictors of outcome on survivors: etiology (Fisher exact test, *p* = 0.003, BF_10_ = 18.9), clinical state that corresponds to clinical diagnosis (Fisher exact test, *p* < 10^−5^, BF_+0_ = 9.7 * 10^6^), and delay since injury (Fisher exact test, *p* < 10^−5^, BF = 2.7 * 10^3^) [see Posner et al. (28) for a recent exhaustive review].

**Figure 2:**
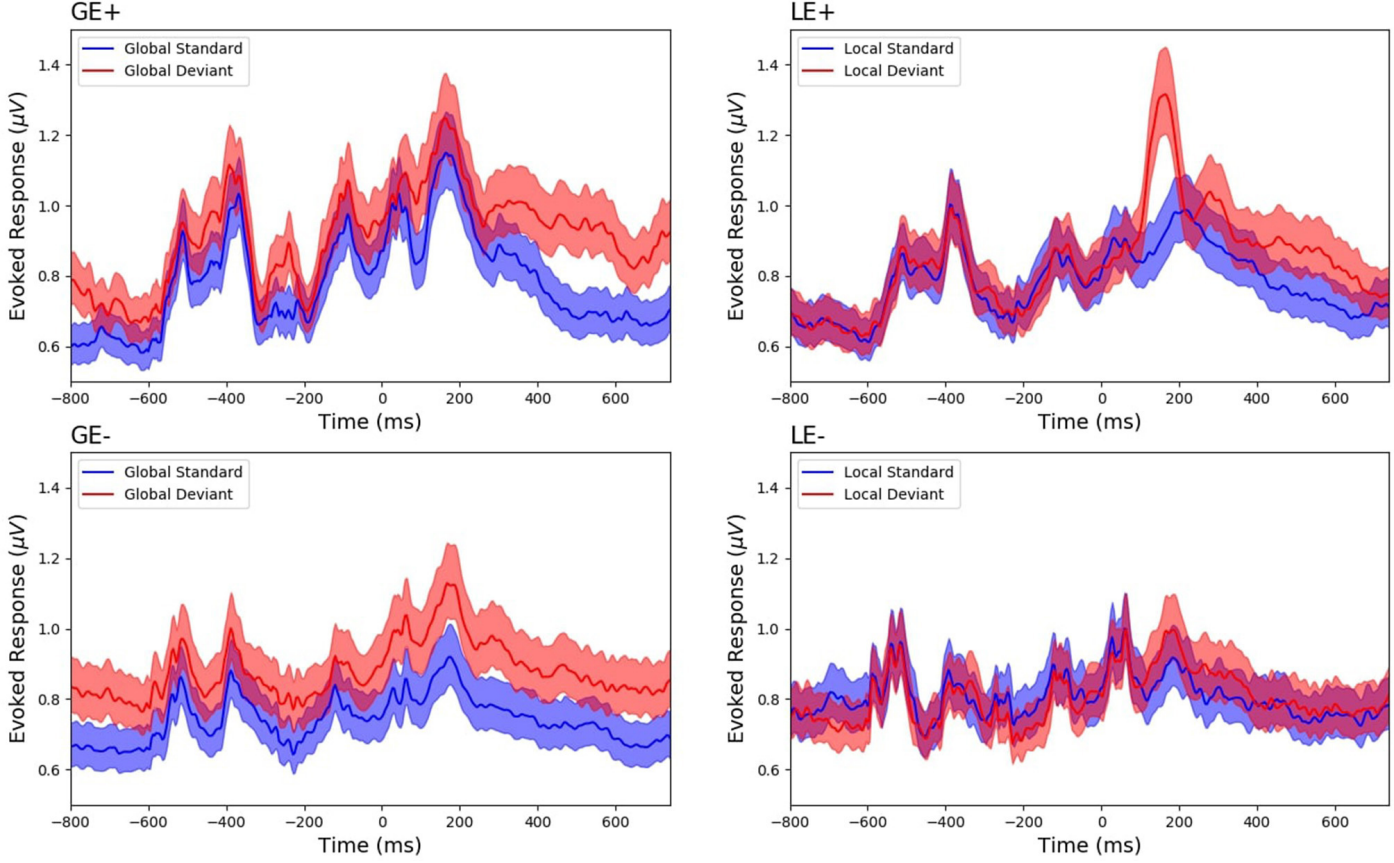
Global field power (GFP) of survivors according to their global effect (GE)(+/–) and local effect (LE)(+/–) status. GFP was computed from mean event-related potentials (ERPs) of deviant (red) and standard (blue) conditions and was plotted with a confidence interval at 95% (shaded areas), respectively, in GE+ patients (*N* = 40; see upper left panel), GE- patients (*N* = 103; see lower left panel), LE+ patients (*N* = 86; see upper right panel), and LE- patients (*N* = 57; see lower right panel). This figure is only shown to illustrate mean ERP patterns: (i) responses to each of the five sounds, (ii) contingent negative variation (CNV) component visible as a ramping ongoing slope in particular in the GE+ group, (iii) mismatch negativity (MMN)-P3a for LE+, and (iv) late P3b component in the GE+ group. No statistical test was calculated given that patients were selected for the corresponding category at the single-subject level statistics.

**Figure 3:**
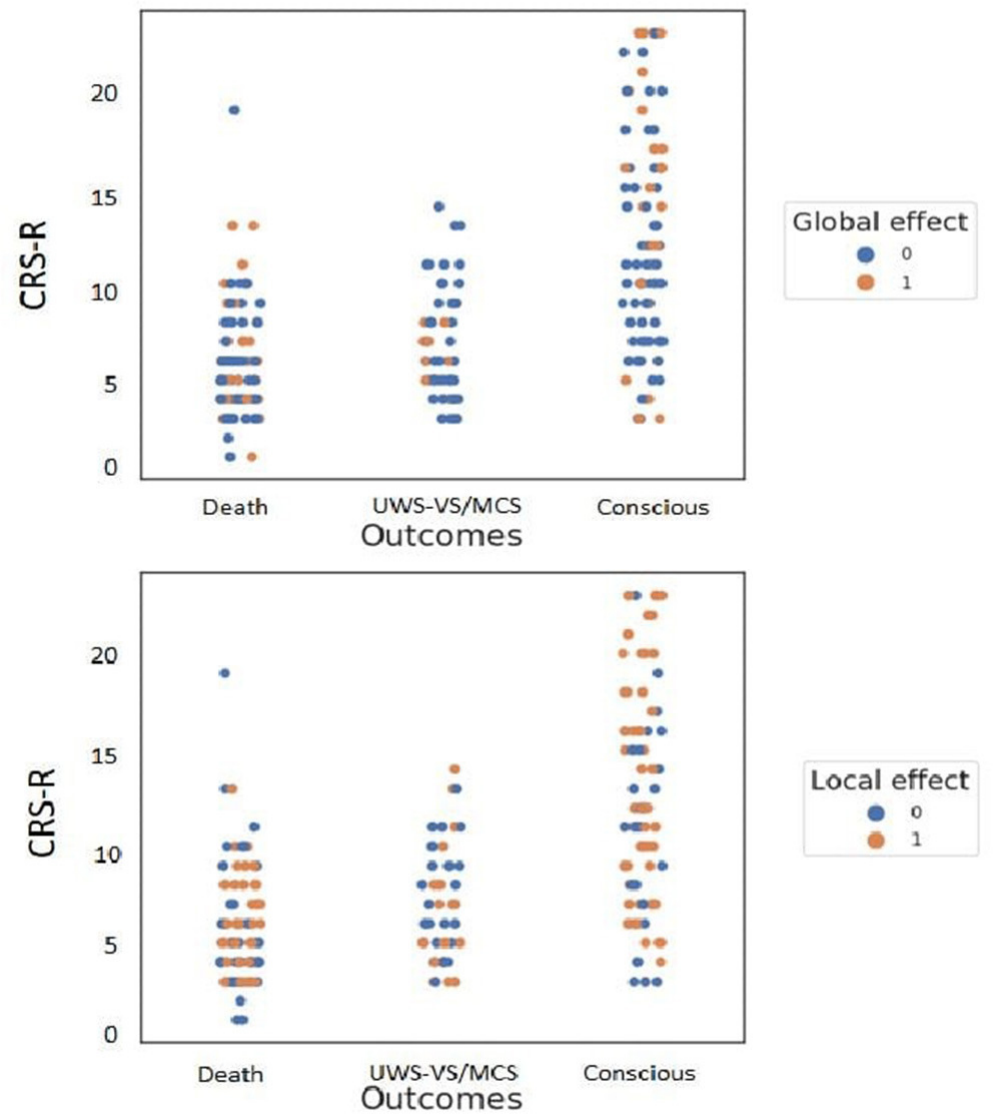
Repartition of patients' outcomes according to their initial clinical state and to global effect (GE) (upper figure) and to local effect (LE) (lower figure) presence. GE was a specific predictor of consciousness recovery in survivors. Note the few GE+ patients with a bad outcome (8/51 = 16%; see upper middle orange dots) contrasting with a higher proportion of LE+ patients with a bad outcome (23/51 = 45%; see lower middle orange dots). Inversely, LE was a sensitive predictor of consciousness recovery. The proportion of LE+ patients who recovered consciousness was larger than the proportion of GE+ with a good outcome (63/92 = 68 vs. 32/92 = 35%; see rightmost upper and lower orange dots).

We then moved to the GE [see Figure 2 for global-field power (GFP) plots of each GE and LE subgroup]. The population of patients with a documented outcome included 66 GE+ patients and 170 GE- patients (Table 1, Supplementary Table 4, Figure 3). The proportion of GE+/GE- patients did not differ between the group of patients with a documented outcome and the group with an unknown outcome (Fisher exact test, *p* = 0.16; BF_+0_ = 0.37).

**Table 1:** Description of patients with a documented outcome in terms of etiology, delay between brain injury and evaluation, and GE status.

	**≤3 months**	**>3 months**
Anoxia	***N*** **= 67**	***N*** **= 17**
	GE+: 15 [5 (E)MCS; 10 VS]	GE+: 3 [2 (E)MCS; 1 VS]
	GE–: 52 [21 (E)MCS; 31 VS]	GE–: 14 [4 (E)MCS; 10 VS]
TBI	**N = 30**	**N = 19**
	GE+: 12 [10 (E)MCS; 2 VS]	GE+: 4 [3 (E)MCS; 1 VS]
	GE–: 18 [11 (E)MCS; 7 VS]	GE–: 15 [10 (E)MCS; 5 VS]
Other	**N = 73**	**N = 30**
	GE+: 21 [14 (E)MCS; 7 VS]	GE+: 11 [9 (E)MCS; 2 VS]
	GE–: 52 [31 (E)MCS; 21 VS]	GE–: 19 [14 (E)MCS; 5 VS]

*(E)MCS, minimally conscious state or exit-MCS; GE, global effect; VS, vegetative state/unresponsive wakefulness syndrome*.

### Predictive Power of Global Effect on Consciousness Recovery in Survivors

We first focused on survivors in order to minimize both the bias of a putative self-fulfilling prophecy that would link GE profile with withdrawing of life-sustaining therapies (WLST) decisions (e.g., more WLST for GE- patients) and to discard the impact of WLST in potentially conscious but extremely impaired patients.

**Figure 4:**
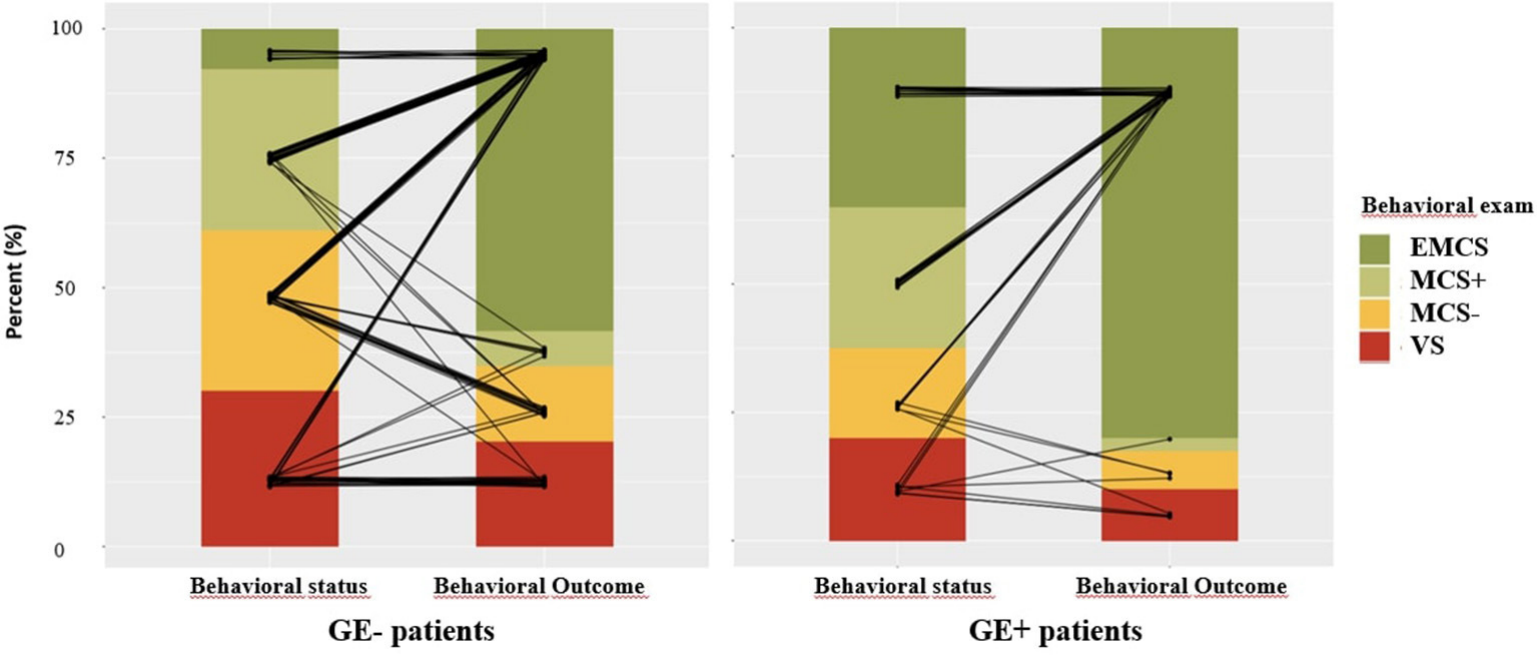
Outcome of global effect (GE)+ and GE– surviving patients. Outcome of GE- patients (left panel) represents each patient by a black segment connected to the patient's initial behavioral status [vegetative state/unresponsive wakefulness syndrome (VS/UWS), minimally conscious state (MCS)–, MCS+, exit-MCS (EMCS)] and to the patient's behavioral outcome (same categories) at 6 months. Outcome of GE+ patients is represented using a similar method in the right panel. Note in particular that all MCS+/GE+ surviving patients recovered overt behavioral evidence of consciousness.

As we hypothesized, GE status predicted consciousness recovery in survivors (Table 2, Supplementary Table 6): the initial presence of a significant GE+ predicted consciousness recovery at 6 months (Fisher exact test, *p* = 0.02) with a high Sp (84%; see Table 2 for 95% CI) and PPV (80%). As previously reported, Se (35%) and NPV (42%) were low. This led to an informative LR+ of 2.22 and a poorly informative LR- of 0.77. Note that the more likelihood ratios depart from 1, the more a test is informative. More specifically, in the present study, an LR+ value superior to 1 informs about the likelihood of consciousness recovery when GE is present, and an LR- value close to 0 informs about the likelihood of no consciousness recovery when GE is absent. A Bayesian analysis (BF_+0_ = 8.85) confirmed the value of GE+ in favor of positive outcome prediction of GE+. Interestingly, 3/8 survivor patients with a GE who did not recover consciousness had a refractory epilepsy with worsening of their neurological status after the initial evaluation, suggesting a deterioration of their cognitive and conscious status after the GE recording. Of special interest, all GE+ MCS+ patients who survived recovered univocal behavioral evidence of consciousness (Figure 4).

**Table 2:** Performance of GE, LE, and clinical status on consciousness recovery in survivors only.

	**GE**	**LE**	**Clinical status**
Se	0.35 [0.25, 0.45]	0.68 [0.58, 0.78]	0.89 [0.81, 0.95]
Sp	0.84 [0.71, 0.93]	0.55 [0.40, 0.69]	0.57 [0.42, 0.71]
PPV	0.80 [0.64, 0.91]	0.73 [0.63, 0.82]	0.79 [0.70, 0.86]
NPV	0.42 [0.32, 0.52]	0.49 [0.36, 0.63]	0.74 [0.58, 0.87]
LR+	2.22 [1.11, 4.44]	1.52 [1.09, 2.12]	2.07 [1.50, 2.85]
LR-	0.77 [0.64, 0.94]	0.57 [0.39, 0.85]	0.19 [0.10-0.36]
BF	8.85	15.97	1.12 × 10^7^

*Numbers indicated in brackets correspond to the 95% confidence interval (CI)*.

*BF, Bayes factor; GE, global effect; LE, local effect; LR-, negative likelihood ratio; LR+, positive likelihood ratio; NPV, negative predictive value; PPV, positive predictive value; Se, sensitivity; Sp, specificity*.

Given the large proportion of GE+ patients in the EMCS group, which corresponds to conscious patients, our results suggested that the presence of a GE could rather confirm the presence of conscious processing in the absence of obvious overt behavioral evidence of consciousness, rather than predicting its later recovery. Indeed, among the 40 GE+ survivor patients with a documented outcome, 14 were initially in an EMCS (35%), whereas 11 were in an MCS+ (27.5%), seven in an MCS- (17.5%), and eight in a VS/UWS (20%). We therefore replicated our main analysis after removing the patients who were in an EMCS during EEG recording. Clearly, GE+ status did not predict recovery of consciousness (Fisher exact test, *p* = 0.26).

Note that the same main analysis (including EMCS patients) performed on the whole population of patients, including dead patients, still showed a similar pattern of results (Supplementary Table 5), but with a less significant effect [*p* = 0.07 and BF_+0_ = 1.9 (anecdotal evidence); Se = 35%; Sp = 76%; PPV = 48%; NVP = 65%; LR+ = 1.47; LR- = 0.85].

### Analyzing the Impact of Deaths

In order to test the potential impact of a self-fulfilling prophecy on survival (i.e., more WSLT for GE- than for GE+ patients), we checked that the proportion of deaths did not differ between GE+ and GE- patients [26/66 (39.4%) vs. 67/170 (39.4%); *p* = 1].

Given that we interpret the presence of a GE as a signature of conscious access, we predicted that GE+ patients were in a behaviorally overt or covert conscious state during the initial EEG recording. Therefore, we explored the cause of death in the GE+ population (*N* = 26; 36.1%): could it be related to secondary degradation that occurred after our evaluation or to other factors independent from the cognitive/consciousness status. Eighteen patients died from WLST including at least five patients who degraded neurologically after initial evaluation and before WLST decision and one patient with severe brainstem lesions with a very poor predicted motor outcome. One patient died in a context of organ donation after cardiac death (Maastricht III). We ignore the cause of death for the eight remaining patients.

### Predictive Power of Initial Clinical Status and Local Effect on Consciousness Recovery

We then computed the outcome prediction value of the initial clinical state and replicated the findings of previous studies (1, 4). When excluding the deaths, initial CRS-R scoring category (VS/UWS vs. MCS or EMCS) indeed predicted consciousness recovery (*p* < 10^−8^; BF_+0_ = 5.6 × 10^14^), but with a different pattern of prediction than GE (Table 2, Figure 3): indeed, while sensitivity (89%), NPV (74%), and LR- (0.19) clearly outperformed the GE (35%, 42%, and 0.77, respectively), the PPV was comparable (79 vs. 80%), and the GE was superior to the CRS-R behavioral scoring in terms of specificity (84 vs. 57%) and LR+ (2.22 vs. 2.07).

Note that similar results were found when keeping the patients who died (*p* < 10^−15^; BF_+0_ = 1.11 × 10^7^; Se = 89%; Sp = 64%; NPV = 90%; PPV = 61%; LR+ = 2.47; LR– = 0.17).

Finally, the ERP LE that indexes non-conscious cortical processing of novelty also predicted consciousness recovery at 6 months in survivors (*p* = 0.007; BF_+0_ = 16.0), with the following pattern: Se (68%), Sp (55%), PPV (73%), NPV (49%), LR+ (1.52), and LR- (0.57). The same analysis while including dead patients replicated this outcome prediction performance (*p* = 0.003; BF_+0_ = 14.6). The same analysis performed after exclusion of EMCS patients showed a trend of an effect (*p* = 0.06).

Interestingly, while the Sp, PPV, and LR+ showed higher numerical values in GE than those in LE, Se, NPV, and LR- were higher in LE than those in GE. This pattern may suggest that while the presence of GE would be predictive of a positive outcome, the absence of LE would be predictive of a bad outcome (LR+ = 2.22 vs. 1.52; LR– = 0.77 vs. 0.57).

## Discussion

The main result of our study reveals the presence of an ERP GE as a specific predictor of behavioral recovery of consciousness. In our study, all EMCS or MCS+ patients with a significant GE recovered a univocal overt behavioral evidence of consciousness. This finding has both clinical and theoretical implications.

Given the large set of evidence showing that GE+ requires conscious processing of the series of sounds and given that the largest category of GE+ patients in the present study corresponded to initially conscious EMCS patients, one may consider the GE as a diagnostic tool to probe consciousness, rather than as a prognosis tool of its later recovery. Indeed, after excluding EMCS patients from the analysis, we could not observe any reliable prognosis value of GE on consciousness recovery in clinically VS/UWS or MCS patients. However, two factors have to be taken into account. First and as noted above, all these patients were addressed to our structure because clinical neurological examination could not determine if they were conscious or not. This was also the case for the EMCS patients: while we could categorize them as conscious after careful and repeated behavioral evaluations using the CRS-R methodology (EMCS category), patients' caregivers including MDs, nurses, and nurse assistant colleagues, as well as patients' relatives could not categorize these patients as being conscious. In contrast, outcome evaluation through our structured phone interview did not require such a subtle expertise, and patients considered as conscious showed univocal and obvious behavioral evidence of consciousness. Therefore, the behavioral status of the GE+ EMCS patients did improve since ERP recording. In other terms, GE+ did not only diagnose consciousness in a specific way, but it also predicted a behavioral improvement of consciousness. Note also that the non-significant trend of an effect in the expected direction after removing initially EMCS patients probably reflects a lack of power. Indeed, simulating a number of patients three times larger effect was sufficient to reach significant values (Fisher exact test, *p* = 0.03). A key question, however, remains unsolved: was this behavioral improvement of consciousness associated with a parallel and equivalent cognitive improvement of consciousness, or was this behavioral improvement occurring under a constant level of preserved consciousness since ERP recording? Under the first hypothesis, one may consider that the presence of a GE reflects the preservation of a necessary but insufficient component of consciousness and therefore constitutes a predictor of behavioral and cognitive improvement. Note however that, obviously, the absence of GE cannot exclude the preservation of such a component. Under the second hypothesis, one would rather consider that the presence of a GE is a direct signature of conscious processing and therefore constitutes a strong diagnostic tool of consciousness and a prognosis tool restricted to behavioral evidence of consciousness. This discussion is clearly beyond the scope of the present report, but the strong relation we discovered between the presence of a GE and the ability to report subjectively global deviance advocates for this second hypothesis (19). Moreover, scalp topography of the ERP GE corresponds to a P3b component (17), and a current scientific debate questions the meaning of P3b: is it a direct neural signature of conscious access, or rather a post-perceptual correlate of cognitive events that are posterior in time to conscious access (29–38)? Interestingly, the single consensual point of this debate consists of considering that P3b presence does require conscious processing (even if it is not the neural signature of conscious access). Within the context, GE presence would rather be interpreted as an evidence for conscious processing of global deviance and, therefore, as an evidence of consciousness during ERP recording.

From a medical perspective, this new marker may improve predictions of cognitive and consciousness recovery in DOC patients. It is noteworthy that a full automatization of the “local–global” task is a realistic objective: delivery of instructions and of auditory stimuli during EEG recording while sending time stamps for each trial, as well as EEG preprocessing and ERP statistics can be fully automatized, including data quality evaluation steps (22). Therefore, while expert behavioral evaluation, which also conveys a strong predictive information of consciousness recovery, is currently limited by a human factor (up to ~40% of behavioral errors, see above), the use of the “local–global” task could be of very valuable help in the absence of human expertise of behavioral examination of DOC patients. Moreover, even after expert and repeated evaluation, we could identify a proportion of ~15% of clinically VS/UWS patients with evidence for conscious processing of the stimuli as indexed by the presence of a GE. This proportion is very close to the ones reported with other functional brain imaging tools and confirms the existence of dissociations between overt behavior and covert cognition and consciousness, even in non-trivial situations such as in the locked-in syndrome (39, 40). The cognitive–motor dissociations (CMDs), as coined by Schiff (13), further confirm the necessity to use such tools in routine when evaluating the current clinical state of patients and when trying to predict their outcome. For instance, we recently reported the presence of a GE in a clinically VS/UWS young patient after severe fat embolism syndrome who then recovered full consciousness and a cognitive and motor autonomy <2 months later (41). Note however that this promising approach also presents limitations. First, 33% of recordings were discarded for data quality issues, mostly related to motor artifacts precluding further analysis of EEG activity. This figure almost replicates our previous report on the diagnosis utility of the “local–global” task (21) in which 35% of recordings had to be discarded. Second, if the GE outperforms clinical evaluation in terms of positive likelihood ration, it clearly has a poor sensitivity and misses many cases of positive outcome. This limitation probably originates from the rational of our approach: not only an individual has to be conscious to show a GE, but he has to perceive auditory stimuli to attend to them, to understand the structure of trials, to keep this information is working memory, and to identify global deviant trials. Understanding task verbal instruction can also be compromised in aphasic patients and may decrease the chances to observe a GE, as shown in our previous comparisons between active explicit and passive attentive versions of the task (17, 19). This multiplication of associated probabilities of each of these conditions may explain the high specificity and poor sensitivity of this marker. Rather than probing directly conscious state, we infer such a state by probing conscious access to a specific attribute of the stimuli (violation of the global rule). This set of necessary conditions may explain why the GE does not discriminate well between clinically MCS and VS/UWS patients, and why only roughly half of conscious patients present such an effect.

While GE proved to be a poorly sensitive (Se = 38%) but very specific (Sp = 84%) marker of overt consciousness recovery, LE also showed a complementary and interesting pattern of predictive power (Se = 68%; Sp = 55%). Previous studies established that the two major components of LE [MMN and P3a ERP components (17)] can be observed both in conscious individuals for unconsciously perceived stimuli and in unconscious individuals [e.g., comatose state (42–44), deep anesthesia (45), sleep (46), and in VS/UWS (17, 20, 21)]. So, the predictive value of consciousness recovery of LE in DOC patients is most probably explained by its value as a marker of residual function preservation of a local cortical network (i.e., auditory cortex and MMN). In other words, the presence of significant LE allows to infer the existence of cortically mediated processes (47). LE presence would therefore play as a necessary but insufficient condition to conscious state, as predicted by several theoretical models such as the global neuronal workspace theory Global Neuronal Workspace Theory (GNWT) (48).

Our findings also speak to biological theories of consciousness. We previously showed that the presence of a GE corresponds to a late (>250 ms after the delivery of the fifth sound) and sustained brain-scale pattern of activity that includes both auditory areas and a distributed frontoparietal network. We interpret this signature of conscious access as the broadcasting of a complex and differentiated representation (49) in a global neuronal workspace (GNW) (48). Here, by revealing that this signature predicts overt recovery of consciousness, our results consolidate the plausibility of such a GNWT.

Finally, most GE+ results were found in conscious EMCS patients. Indeed, MCS and VS/UWS patients showed the same proportion of GE+ recordings, as previously reported on a smaller population of patients (21). These two findings support the recent reinterpretation of MCS category (47) as a cortically mediated state (CMS) that informs us with certitude about the behaviorally overt residual integrity of some cortical networks, but that does not speak univocally about an elusive conscious state. In sharp difference with VS/UWS, whose behavioral items do not recruit cortical networks (e.g., auditory startle is a brainstem-mediated behavior), each of the MCS items of the CRS-R solicits a specialized cortical network (e.g., visual pursuit requires an occipital-parieto-FEF cortical network) and therefore reveals its functional integrity. As a consequence, and given that multiple examples of cortical unconscious processing has been shown in conscious and in unconscious individuals, the links prevailing between MCS and consciousness is probably less strong than the MCS acronym states and suggests. Indeed, while cortical processing is a necessary condition for consciousness (*all conscious individuals have cortical processing*), this generic condition is not sufficient (*many unconscious individuals still have some forms of cortical processing*). In other terms, if MCS rather indexes a CMS, it is indeed expected to be associated with an overall better prognosis of consciousness recovery, but not as strongly and clearly as suggested by its name. This is precisely why additional prognosis markers, such as the GE, may be of prime interest.

## Data Availability Statement

The original contributions generated for the study are included in the article/Supplementary Material, further inquiries can be directed to the corresponding author/s.

## Ethics Statement

The studies involving human participants were reviewed and approved by CPP IDF1 Ethical committee. Written informed consent for participation was not required for this study in accordance with the national legislation and the institutional requirements.

## Author Contributions

PP, BR, JS, and LN contributed to the study concept and design. PP, BH, MV, FF, and BR contributed to the data collection. PP, BR, JS, and LN contributed to the analysis and interpretation of data. PP and LN contributed to the drafting of the manuscript, statistical analysis, had full access to all the data in the study, took responsibility for the integrity of the data, and the accuracy of the data analysis. All authors contributed to the article and approved the submitted version.

## Conflict of Interest

The authors declare that the research was conducted in the absence of any commercial or financial relationships that could be construed as a potential conflict of interest.

## Abbreviations

CRS-R: Coma Recovery Scale-Revised
DOC: disorder of consciousness
EEG: electroencephalography
ERP: event-related potential
GNW: global neuronal workspace
GE+/–: presence/absence of global effect
LE+/–: presence/absence of local effect
LR+: positive likelihood ratio
LR–: negative likelihood ratio
MCS: minimally conscious state
NPV: negative predictive value
PPV: positive predictive value
Sp: specificity
Se: sensibility
VS/UWS: vegetative state/unresponsive wakefulness syndrome.
