# Supplementary material for: Auditory Event-Related “Global Effect” Predicts Recovery of Overt Consciousness
Source: Front Neurol. 2021 Jan 8;11:588233. doi: 10.3389/fneur.2020.588233 (PMC7819971; doi:10.3389/fneur.2020.588233)
Supplement: Supplementary file 1 [file Data_Sheet_1.PDF]

Patient numb	Gender	Age	Etiology	Delay	Diagnostic	CRS-R	Outcome	Global effect	Local effect	Etiologic details
1	M	<45 years	TBI	Chronic	MCS+	20	Conscious	1	1	-
2	M	<45 years	Other	Acute	VS	5	UWS-VS	0	1	ADEM
3	F	>45 years	Anoxia	Acute	VS	3	UWS-VS	0	1	-
4	F	>45 years	Other	Acute	VS	4	Death	0	0	Subarachnoid hemmorhage
5	M	<45 years	Other	Chronic	MCS-	8	Death	0	1	Cerebral hematoma
6	M	<45 years	TBI	Acute	EMCS	19	Conscious	1	0	-
7	M	<45 years	TBI	Chronic	VS	5	-	0	1	-
8	F	>45 years	Other	Acute	VS	6	Death	0	0	Stroke per cardiac surgery
9	M	<45 years	Anoxia	Chronic	VS	4	UWS-VS	0	0	-
10	M	<45 years	TBI	Chronic	VS	6	Conscious	0	1	-
11	M	<45 years	Other	Chronic	EMCS	12	Conscious	1	1	Cerebral hematoma
12	M	<45 years	Anoxia	Acute	VS	3	Death	0	0	-
13	M	>45 years	TBI	Acute	MCS+	13	-	0	1	-
14	F	<45 years	Anoxia	Acute	VS	5	UWS-VS	0	1	-
15	M	>45 years	Anoxia	Acute	VS	4	Death	0	0	-
16	M	>45 years	Anoxia	Chronic	MCS-	14	MCS	0	1	-
17	F	>45 years	Other	Chronic	MCS+	9	Death	1	0	Malignant hyperthermia
18	M	>45 years	TBI	Acute	MCS-	11	Conscious	0	1	-
19	F	<45 years	Anoxia	Chronic	MCS+	6	Death	0	0	-
20	M	<45 years	Anoxia	Acute	MCS+	13	Conscious	0	0	-
21	M	>45 years	Anoxia	Acute	VS	4	Death	0	0	-
22	M	>45 years	Anoxia	Acute	VS	4	-	0	0	-
23	M	>45 years	Other	Acute	MCS+	10	Conscious	0	1	Stroke
24	M	>45 years	Anoxia	Acute	MCS+	19	Conscious	1	1	-
25	M	>45 years	Anoxia	Acute	EMCS	10	-	0	1	-
26	M	<45 years	Anoxia	Acute	VS	3	Death	0	0	-
27	M	<45 years	TBI	Acute	VS	6	Death	0	0	-
28	M	<45 years	TBI	Chronic	MCS-	9	UWS-VS	0	0	-
29	F	<45 years	Anoxia	Acute	VS	1	Death	0	0	-
30	M	>45 years	Other	Acute	MCS-	11	Conscious	0	0	Drug intoxication
31	F	<45 years	TBI	Chronic	MCS+	8	MCS	0	0	-
32	M	>45 years	Anoxia	Acute	VS	4	UWS-VS	1	1	-
33	M	>45 years	Anoxia	Acute	VS	5	Death	0	0	-
34	F	>45 years	TBI	Chronic	MCS-	8	MCS	1	1	-
35	F	<45 years	Anoxia	Chronic	VS	6	UWS-VS	0	0	-
36	F	<45 years	TBI	Acute	MCS-	10	Conscious	0	1	-
37	M	<45 years	Anoxia	Chronic	VS	5	Death	1	0	-
38	F	>45 years	Other	Chronic	VS	5	UWS-VS	0	0	Drug intoxication
39	M	<45 years	Other	Acute	VS	4	MCS	0	0	Encephalitis of unknown cause

40	M	>45 years	Other	Acute	MCS+	10	Conscious	0	1	Cardiac surgery complication
41	M	>45 years	Other	Acute	MCS-	7	MCS	0	1	Hypoglycemia
42	M	>45 years	Other	Acute	VS	6	Death	1	1	Encephalitis of unknown cause
43	M	<45 years	TBI	Chronic	VS	8	MCS	0	1	-
44	F	<45 years	Other	Acute	MCS+	16	Conscious	0	0	Encephalitis post graft
45	M	>45 years	Anoxia	Acute	MCS+	11	Death	1	0	-
46	M	<45 years	TBI	Acute	VS	6	Death	0	0	-
47	F	>45 years	Other	Acute	MCS-	10	Death	0	0	Septic encephalitis
48	F	>45 years	Other	Chronic	MCS-	9	MCS	0	0	Stroke
49	M	<45 years	Anoxia	Acute	VS	4	Death	1	0	-
50	M	<45 years	Anoxia	Acute	VS	5	UWS-VS	0	0	-
51	F	<45 years	Other	Chronic	MCS+	14	Conscious	0	0	Subarachnoid hemmorhage
52	F	<45 years	Other	Acute	VS	4	Death	0	0	Hypoglycemia
53	M	<45 years	TBI	Acute	VS	5	-	0	0	-
54	M	>45 years	Other	Acute	EMCS	20	Conscious	0	1	Septic encephalitis
55	M	<45 years	TBI	Acute	MCS-	7	Conscious	0	0	-
56	F	>45 years	Other	Acute	MCS-	8	MCS	0	0	Cerebral hematoma
57	F	<45 years	Anoxia	Acute	MCS-	10	MCS	0	0	-
58	M	>45 years	Other	Chronic	MCS+	5	Death	0	1	Craniopharyngioma
59	M	>45 years	Other	Acute	VS	4	-	0	1	Stroke per cardiac surgery
60	F	<45 years	TBI	Chronic	MCS-	8	-	0	0	-
61	F	<45 years	TBI	Chronic	MCS+	13	-	0	1	-
62	F	<45 years	Anoxia	Acute	VS	3	-	0	1	-
63	M	>45 years	TBI	Chronic	MCS-	10	MCS	0	1	-
64	M	<45 years	TBI	Acute	VS	6	MCS	1	0	-
65	M	<45 years	Anoxia	Acute	VS	3	Conscious	0	0	-
66	F	<45 years	Other	Chronic	MCS-	7	Conscious	0	1	Hydrocephalus
67	M	>45 years	TBI	Acute	MCS+	12	Conscious	1	0	-
68	F	>45 years	Other	Acute	MCS-	10	Conscious	0	1	Cerebral thrombosis
69	F	>45 years	Anoxia	Acute	MCS-	13	MCS	0	0	-
70	M	>45 years	Other	Chronic	MCS+	14	Conscious	0	1	Status epilepticus
71	M	>45 years	Other	Chronic	MCS+	13	Conscious	0	0	Subarachnoid hemmorhage
72	M	>45 years	Other	Acute	VS	6	Death	1	1	Encephalitis of unknown cause
73	M	>45 years	Other	Acute	MCS+	8	Death	0	1	Srtoke
74	M	<45 years	TBI	Chronic	MCS-	10	MCS	0	0	-
75	F	>45 years	Other	Chronic	EMCS	22	-	0	1	Meningioma
76	M	<45 years	Other	Chronic	EMCS	14	Conscious	1	1	Creutzfeldt Jakob disease
77	F	>45 years	Anoxia	Acute	VS	5	Death	0	0	-
78	M	>45 years	TBI	Chronic	VS	7	-	0	0	-
79	M	>45 years	Other	Acute	VS	5	Death	0	0	Autoimmune encephalitis

80	F	>45 years	Other	Acute	MCS-	8	Death	0	1	Encephalitis of unknown cause
81	M	>45 years	Other	Acute	MCS-	3	-	0	0	Acute polyradiculoneuritis
82	M	>45 years	Anoxia	Chronic	MCS+	13	Death	1	0	-
83	M	>45 years	Anoxia	Acute	VS	4	Death	0	1	-
84	M	<45 years	Other	Acute	MCS-	10	Death	1	1	Toxoplasmosis
85	M	>45 years	Anoxia	Acute	VS	8	Death	1	1	-
86	M	>45 years	Other	Acute	EMCS	11	Conscious	0	1	Thrombotic microangiopathy
87	F	<45 years	Other	Chronic	VS	4	Conscious	0	0	Anti NMDAR encephalitis
88	M	>45 years	Other	Chronic	MCS+	5	Conscious	0	1	Subarachnoid hemmorhage
89	M	>45 years	Other	Acute	MCS-	6	Death	0	1	Hypoglycemia
90	F	<45 years	Anoxia	Acute	VS	4	Death	0	0	-
91	M	>45 years	TBI	Acute	VS	3	Conscious	0	0	-
92	M	>45 years	Other	Acute	VS	4	Death	0	0	Septic encephalitis
93	F	<45 years	Anoxia	Acute	MCS+	11	UWS-VS	0	0	-
94	M	>45 years	Other	Acute	VS	5	Death	1	1	Septic encephalitis
95	F	>45 years	Anoxia	Acute	VS	5	UWS-VS	0	0	-
96	F	<45 years	TBI	Chronic	VS	7	UWS-VS	1	0	-
97	F	<45 years	Other	Acute	EMCS	16	Conscious	0	1	Meningitidis
98	M	>45 years	Anoxia	Acute	VS	4	Death	0	0	-
99	M	<45 years	Anoxia	Acute	VS	5	Death	0	0	-
100	F	<45 years	Anoxia	Acute	MCS-	9	Conscious	0	0	-
101	M	>45 years	Anoxia	Acute	VS	6	Death	0	0	-
102	F	>45 years	Anoxia	Acute	MCS-	9	-	0	1	-
103	M	<45 years	Anoxia	Acute	MCS+	10	Death	0	1	-
104	F	<45 years	Anoxia	Acute	MCS-	7	Conscious	0	1	-
105	F	>45 years	Other	Acute	EMCS	17	Conscious	1	0	Cerebral hematoma
106	M	>45 years	Other	Acute	MCS-	9	Death	0	1	Cerebral hematoma
107	F	>45 years	Anoxia	Acute	MCS-	6	MCS	0	1	-
108	M	<45 years	Anoxia	Acute	MCS-	11	MCS	0	1	-
109	M	>45 years	Anoxia	Chronic	VS	4	MCS	0	1	-
110	F	>45 years	Other	Chronic	MCS-	13	MCS	0	1	Cerebral hematoma
111	M	>45 years	Anoxia	Chronic	VS	6	Death	0	0	-
112	M	<45 years	TBI	Chronic	MCS-	9	MCS	0	0	-
113	M	>45 years	TBI	Acute	MCS-	6	-	0	0	-
114	F	>45 years	Other	Chronic	MCS-	8	-	0	1	Cerebral hematoma
115	F	<45 years	Other	Acute	EMCS	17	Conscious	1	1	Subarachnoid hemmorhage
116	M	<45 years	Anoxia	Acute	MCS-	8	Conscious	0	0	-
117	M	>45 years	Anoxia	Acute	MCS-	6	Conscious	0	1	-
118	F	>45 years	Anoxia	Chronic	VS	6	Death	0	1	-
119	F	>45 years	Anoxia	Chronic	VS	5	UWS-VS	0	0	-

120	M	>45 years	Other	Acute	MCS+	15	Conscious	1	1	Hypoglycemia
121	M	>45 years	Other	Acute	VS	3	Death	0	1	Anti NMDAR encephalitis
122	F	<45 years	Anoxia	Chronic	VS	4	UWS-VS	0	0	-
123	F	>45 years	Other	Acute	VS	5	Death	0	1	Cerebral hematoma
124	M	<45 years	Other	Acute	MCS-	7	Conscious	0	1	Septic encephalitis
125	F	<45 years	TBI	Acute	MCS+	13	Conscious	1	0	-
126	M	>45 years	Anoxia	Acute	VS	5	-	0	0	-
127	M	<45 years	TBI	Acute	VS	6	-	1	1	-
128	M	>45 years	Anoxia	Acute	EMCS	22	Conscious	0	1	-
129	F	>45 years	Other	Acute	VS	5	-	1	1	Autoimmune encephalitis
130	M	<45 years	Other	Acute	MCS-	10	Conscious	0	0	Cerebral hematoma
131	M	<45 years	Anoxia	Acute	VS	5	Conscious	0	1	-
132	M	<45 years	Other	Acute	MCS+	10	Death	1	1	Cerebral hematoma
133	M	<45 years	TBI	Acute	MCS+	12	Conscious	0	1	-
134	M	<45 years	TBI	Acute	MCS+	12	Conscious	1	1	-
135	M	<45 years	Other	Chronic	EMCS	13	-	1	1	Status epilepticus
136	M	<45 years	TBI	Chronic	VS	5	MCS	0	1	-
137	F	>45 years	Anoxia	Acute	VS	3	Death	0	0	-
138	F	>45 years	Anoxia	Acute	VS	3	Death	0	0	-
139	F	<45 years	Other	Acute	MCS+	6	-	0	1	Stroke
140	M	>45 years	Other	Chronic	VS	5	-	0	1	Hypertensive encephalopathy
141	M	<45 years	TBI	Acute	MCS-	8	-	0	1	-
142	M	>45 years	Anoxia	Acute	VS	4	Death	1	1	-
143	M	<45 years	Other	Acute	MCS+	16	Conscious	0	1	Stroke
144	F	<45 years	Other	Acute	VS	5	-	1	1	Encephalitis of unknown cause
145	M	>45 years	TBI	Acute	MCS+	8	-	0	1	-
146	M	>45 years	Anoxia	Acute	VS	5	Death	0	1	-
147	M	>45 years	Other	Acute	MCS-	8	Death	0	1	Stroke
148	M	<45 years	Other	Acute	MCS-	14	-	0	0	Drug intoxication
149	M	<45 years	Anoxia	Acute	MCS+	8	Conscious	0	0	-
150	M	<45 years	Anoxia	Acute	EMCS	21	-	0	1	-
151	M	>45 years	Other	Chronic	MCS+	16	-	0	1	Cerebral hematoma
152	M	<45 years	Other	Acute	MCS-	8	-	0	0	Cerebral hematoma
153	M	>45 years	Anoxia	Chronic	VS	5	UWS-VS	0	0	-
154	F	<45 years	TBI	Acute	MCS-	7	Conscious	0	1	-
155	M	<45 years	TBI	Acute	VS	7	Conscious	0	1	-
156	F	<45 years	Other	Chronic	VS	4	UWS-VS	0	0	Cerebral hematoma
157	F	<45 years	Anoxia	Acute	VS	4	Death	0	0	-
158	M	<45 years	TBI	Acute	VS	6	Death	0	1	-
159	F	<45 years	Anoxia	Acute	VS	4	Death	0	0	-

160	M	<45 years	Anoxia	Acute	MCS-	9	-	1	1	-
161	M	<45 years	TBI	Chronic	EMCS	18	Conscious	0	1	-
162	M	<45 years	Other	Chronic	VS	5	-	0	0	Subarachnoid hemmorhage
163	M	>45 years	Other	Acute	MCS+	9	Death	1	0	Status epilepticus
164	M	<45 years	Other	Acute	VS	5	-	0	0	Cerebral hematoma
165	M	>45 years	Other	Acute	VS	6	Death	0	1	Encephalitis of unknown cause
166	M	<45 years	Other	Acute	VS	6	Death	0	1	Septic encephalitis
167	M	>45 years	Anoxia	Acute	VS	3	-	0	1	-
168	F	>45 years	Other	Acute	MCS+	10	-	0	0	Encephalitis of unknown cause
169	M	>45 years	Other	Acute	MCS-	8	-	1	1	Subarachnoid hemmorhage
170	-	>45 years	Anoxia	Chronic	EMCS	23	Conscious	1	1	-
171	M	<45 years	Other	Chronic	EMCS	23	Conscious	1	1	Autoimmune encephalitis
172	M	<45 years	Other	Acute	MCS+	6	Conscious	0	1	Stroke
173	M	<45 years	TBI	Chronic	MCS+	7	-	0	1	-
174	M	<45 years	Other	Chronic	EMCS	23	Conscious	1	0	Drug intoxication
175	F	<45 years	Other	Acute	VS	3	Conscious	1	0	Cerebral hematoma
176	M	>45 years	Anoxia	Acute	VS	3	-	0	0	-
177	F	<45 years	Other	Acute	MCS+	18	Conscious	0	1	Hypoglycemia
178	F	<45 years	Anoxia	Chronic	VS	4	-	0	0	-
179	F	>45 years	Other	Acute	VS	3	-	1	1	Cerebral hematoma
180	M	>45 years	Other	Chronic	MCS+	10	-	1	1	Subarachnoid hemmorhage
181	F	>45 years	Other	Acute	VS	7	UWS-VS	0	1	Subarachnoid hemmorhage
182	M	<45 years	TBI	Acute	VS	4	-	1	1	-
183	M	>45 years	Other	Acute	VS	4	Death	0	1	Cerebral hematoma
184	F	>45 years	Other	Chronic	EMCS	20	Conscious	0	1	Subarachnoid hemmorhage
185	F	>45 years	Other	Acute	VS	1	-	0	1	ADEM
186	F	>45 years	Anoxia	Acute	VS	4	Death	0	0	-
187	M	>45 years	Other	Chronic	EMCS	21	-	0	0	Cerebral hematoma
188	M	<45 years	Anoxia	Acute	MCS+	9	Conscious	0	0	-
189	M	>45 years	Other	Acute	VS	6	Death	0	0	Hypoglycemia
190	M	>45 years	Anoxia	Acute	MCS+	15	Conscious	0	1	-
191	M	<45 years	TBI	Acute	MCS+	11	Conscious	0	0	-
192	M	>45 years	Other	Acute	MCS+	8	Death	0	1	Septic encephalitis
193	F	>45 years	Anoxia	Acute	VS	8	-	0	1	-
194	M	<45 years	Anoxia	Acute	VS	3	UWS-VS	0	1	-
195	M	<45 years	Other	Chronic	VS	6	-	1	0	Subarachnoid hemmorhage
196	M	>45 years	Other	Acute	EMCS	16	Conscious	1	1	Acute polyradiculoneuritis
197	F	<45 years	Other	Chronic	VS	3	Death	0	1	Anti NMDAR encephalitis
198	M	<45 years	TBI	Acute	MCS-	12	Conscious	0	0	-
199	M	>45 years	Other	Acute	MCS+	12	Conscious	1	1	Stroke

200	M	>45 years	Other	Acute	MCS+	9	Death	0	1 Stroke
201	M	<45 years	Anoxia	Acute	VS	4	-	0	1 -
202	M	>45 years	Anoxia	Acute	MCS+	10	Conscious	0	1 -
203	F	<45 years	Other	Chronic	EMCS	23	Conscious	1	1 Septic encephalitis
204	F	<45 years	TBI	Chronic	VS	7	-	0	0 -
205	M	>45 years	Anoxia	Acute	EMCS	23	Conscious	1	1 -
206	M	<45 years	Anoxia	Chronic	VS	3	UWS-VS	0	0 -
207	F	>45 years	Anoxia	Acute	VS	4	Death	1	0 -
208	M	>45 years	Anoxia	Acute	MCS+	13	Death	1	1 -
209	M	>45 years	Other	Acute	MCS+	6	Conscious	0	0 Cerebral hematoma
210	F	>45 years	Anoxia	Acute	MCS+	19	Death	0	0 -
211	M	<45 years	TBI	Acute	VS	6	-	0	1 -
212	M	<45 years	Anoxia	Chronic	MCS+	11	Conscious	0	0 -
213	F	<45 years	TBI	Acute	MCS-	9	Conscious	1	1 -
214	M	>45 years	Other	Acute	MCS-	7	Death	0	1 Meningitidis
215	M	<45 years	TBI	Acute	MCS-	9	Conscious	0	1 -
216	M	<45 years	Other	Chronic	EMCS	22	Conscious	0	1 Stroke
217	M	>45 years	Anoxia	Acute	VS	5	-	0	1 -
218	M	>45 years	Other	Acute	VS	6	Conscious	0	1 Central pontine myelinosis
219	M	>45 years	Other	Acute	MCS+	15	Conscious	0	0 Cerebral hematoma
220	M	<45 years	TBI	Acute	VS	3	Death	0	1 -
221	F	<45 years	TBI	Chronic	MCS+	16	-	0	1 -
222	F	<45 years	TBI	Chronic	VS	5	-	1	0 -
223	F	>45 years	Other	Chronic	EMCS	21	-	0	1 Cerebral hematoma
224	F	<45 years	Other	Acute	VS	7	Death	1	0 Cerebral hematoma
225	F	>45 years	Other	Acute	MCS-	7	Death	1	1 Autoimmune encephalitis
226	M	>45 years	Other	Acute	MCS+	11	-	0	0 Stroke
227	F	>45 years	Anoxia	Acute	MCS+	10	-	0	0 -
228	M	>45 years	TBI	Chronic	MCS+	12	Conscious	0	1 -
229	F	<45 years	TBI	Acute	MCS-	8	Conscious	0	1 -
230	M	>45 years	Other	Acute	EMCS	21	Conscious	1	1 Cerebral lymphoma
231	F	>45 years	Other	Acute	MCS-	8	-	0	1 Encephalitis of unknown cause
232	F	<45 years	Other	Acute	VS	5	-	0	1 stroke
233	M	>45 years	Other	Acute	MCS+	9	Conscious	0	1 Cerebral hematoma
234	M	<45 years	Other	Acute	EMCS	16	Conscious	1	1 Cerebral hematoma
235	M	<45 years	Other	Acute	VS	6	-	0	1 Cerebral hematoma
236	M	<45 years	Anoxia	Acute	VS	2	Death	0	0 -
237	M	>45 years	Other	Acute	VS	6	Conscious	1	1 Cerebral thrombosis
238	M	<45 years	TBI	Chronic	EMCS	15	-	0	1 -
239	M	>45 years	TBI	Acute	EMCS	16	Conscious	1	1 -

240	M	>45 years	Other	Acute	VS	3	Death	0	0	Stroke
241	M	>45 years	Anoxia	Acute	MCS+	10	Conscious	0	1	-
242	M	<45 years	Other	Acute	MCS+	3	Conscious	1	1	Acute polyradiculoneuritis
243	M	<45 years	Anoxia	Acute	VS	7	UWS-VS	1	1	-
244	M	>45 years	Anoxia	Acute	VS	3	Death	1	1	-
245	M	>45 years	Other	Acute	VS	4	Death	0	0	Encephalitis post graft
246	M	>45 years	Anoxia	Acute	VS	4	-	0	1	-
247	F	<45 years	Anoxia	Chronic	EMCS	23	Conscious	0	1	-
248	M	<45 years	Anoxia	Acute	MCS+	10	Conscious	0	1	-
249	M	>45 years	Other	Acute	VS	6	Death	0	1	Stroke
250	M	<45 years	Other	Acute	VS	3	-	1	1	Organophosphate intoxication
251	M	>45 years	Anoxia	Acute	MCS+	15	Conscious	1	1	-
252	M	<45 years	TBI	Acute	MCS-	8	-	0	0	-
253	M	>45 years	Anoxia	Acute	VS	7	-	0	1	-
254	F	<45 years	Anoxia	Acute	VS	4	Death	0	1	-
255	M	<45 years	Other	Chronic	VS	5	-	0	0	Encephalitis of unknown cause
256	M	<45 years	TBI	Chronic	MCS+	17	-	1	1	-
257	F	<45 years	Other	Acute	MCS+	15	Conscious	0	0	Catatonia
258	M	<45 years	Other	Acute	MCS-	10	Death	0	0	Cerebral hematoma
259	M	>45 years	TBI	Acute	MCS-	6	Death	0	1	-
260	M	>45 years	TBI	Acute	VS	4	-	0	1	-
261	M	>45 years	Other	Acute	VS	3	-	0	1	Encephalitis of unknown cause
262	M	<45 years	TBI	Acute	MCS+	11	Conscious	1	1	-
263	M	>45 years	Anoxia	Chronic	VS	6	UWS-VS	0	0	-
264	M	<45 years	TBI	Acute	MCS-	10	Conscious	1	1	-
265	F	>45 years	Other	Acute	VS	4	Death	1	1	Cerebral hematoma
266	F	>45 years	Other	Acute	MCS-	10	-	0	1	Cerebral hematoma
267	M	>45 years	Other	Acute	VS	3	Death	0	1	Cerebral hematoma
268	M	<45 years	Anoxia	Acute	VS	4	-	0	1	-
269	F	>45 years	Other	Acute	MCS+	18	Conscious	0	1	Cerebral hematoma
270	F	>45 years	Other	Chronic	MCS-	7	Death	0	0	Hypoglycemia
271	M	<45 years	TBI	Acute	MCS-	5	Conscious	1	1	-
272	M	>45 years	Anoxia	Acute	VS	3	Death	0	0	-
273	M	>45 years	Anoxia	Acute	VS	4	Death	1	1	-
274	M	>45 years	Other	Acute	VS	6	MCS	0	0	Subarachnoid hemmorhage
275	M	>45 years	Other	Chronic	MCS+	14	Conscious	1	1	Stroke
276	F	>45 years	Anoxia	Acute	VS	5	Death	0	1	-
277	M	>45 years	Anoxia	Acute	VS	3	Death	1	1	-
278	M	<45 years	Other	Acute	MCS-	4	Conscious	1	1	ADEM
279	M	>45 years	Other	Acute	MCS+	1	Death	1	0	Stroke of brainstem

280	F	>45 years	TBI	Acute	MCS-	11	-	0	0	-
281	M	<45 years	TBI	Chronic	MCS-	9	MCS	0	0	-
282	F	<45 years	TBI	Acute	MCS-	7	Death	1	1	-
283	M	>45 years	TBI	Chronic	MCS+	11	MCS	0	0	-
284	M	>45 years	Anoxia	Acute	VS	3	Death	1	1	-
285	M	>45 years	Other	Acute	MCS+	16	Conscious	0	0	Cerebral hematoma
286	F	<45 years	TBI	Chronic	MCS-	8	MCS	1	1	-
287	F	<45 years	Anoxia	Acute	MCS-	10	Death	0	0	-
288	M	<45 years	TBI	Acute	VS	6	MCS	1	0	-
289	M	>45 years	Other	Acute	MCS-	5	-	0	0	Toxic encephalitis
290	F	>45 years	Other	Acute	VS	3	Death	0	0	Septic encephalitis
291	F	>45 years	Other	Chronic	VS	5	-	1	0	Subarachnoid hemmorhage
292	M	<45 years	TBI	Acute	MCS-	7	Conscious	0	0	-
293	M	<45 years	Other	Chronic	MCS-	7	Death	1	1	Hypoglycemia
294	F	<45 years	Other	Chronic	VS	4	Conscious	1	0	Anti NMDAR encephalitis
295	F	>45 years	Other	Chronic	MCS-	5	UWS-VS	1	1	Progressive multifocal leukoencephalopathy
296	M	>45 years	TBI	Chronic	EMCS	18	-	0	0	-
297	F	>45 years	Anoxia	Acute	VS	3	Death	0	1	-
298	F	>45 years	Other	Acute	MCS+	19	-	0	1	Subarachnoid hemmorhage
299	F	>45 years	Other	Acute	MCS-	7	-	0	1	Drug intoxication
300	M	>45 years	TBI	Acute	VS	3	MCS	0	1	-
301	M	<45 years	Other	Chronic	VS	5	Death	1	1	ADEM
302	M	<45 years	TBI	Chronic	VS	5	UWS-VS	0	1	-
303	F	>45 years	Other	Chronic	MCS+	20	Conscious	0	1	Subarachnoid hemmorhage
304	M	<45 years	TBI	Chronic	VS	5	UWS-VS	0	1	-
305	M	<45 years	TBI	Chronic	MCS-	4	MCS	0	1	-
306	M	<45 years	Anoxia	Acute	VS	4	Death	0	0	-
307	F	>45 years	Anoxia	Acute	MCS+	9	Death	0	1	-
308	M	<45 years	Other	Chronic	MCS+	11	MCS	0	0	Tuberculous meningoencephalitis
309	F	<45 years	Other	Chronic	VS	3	Death	0	1	Encephalitis of unknown cause
