# Supplementary material for: Auditory Event-Related “Global Effect” Predicts Recovery of Overt Consciousness
Source: Front Neurol. 2021 Jan 8;11:588233. doi: 10.3389/fneur.2020.588233 (PMC7819971; doi:10.3389/fneur.2020.588233)
Supplement: Supplementary file 2 [file Data_Sheet_2.PDF]

Supplementary Material

Supplementary Methods

Series of five complex 50ms-duration sounds were presented via headphones with an intensity of 70dB and 150ms SOA (stimulus onset asynchrony) between sounds. Each sound was composed of three sinusoidal tones (either 350 700 and 1400 Hz, hereafter sound A; or 500 Hz 1000 Hz and 2000 Hz, hereafter sound B). All tones were prepared with 7 ms rise and 7 ms fall times. Four different series of sounds were used, the first two using the same five sounds (AAAAA or BBBBB); and the second with the final sound swapped (either AAAAB or BBBBA). Series of sounds were separated by a variable interval of 1350ms to 1650ms (50ms steps). All subjects heard eight blocks (3-4 minutes duration), in randomized order for each subject (each of the four possible block types was presented twice). The blocks were designed to contain the sound series with a different sound in the end, either as an infrequent stimulus (block type a: 80% AAAAA / 20% AAAAB; block type b: 80% BBBBB / 20% BBBBA); or as a frequent stimulus (block type c: 80% AAAAB / 20% AAAAA; block type d: 80% BBBBA / 20% BBBBB). All block types presented a local regularity (the fifth sound could be different or identical to previous sounds) and a global regularity (one of the series of sounds was less frequent than the other). Each block started with 20-30 frequent series of sounds to establish the global regularity before the first infrequent stimulus arrival. In each block the number of infrequent trials varied between 22 and 30. We used the active counting version of the local-global task: instructions were auditory delivered at the beginning of each block as follow: “You will now listen to repetitive series of 5 sounds. At the beginning, series are all the same and define ’the rule’. In a second time, some series will be different from the first ones. When you will hear such a series, different from the rule, we ask you to pay attention to it and to count it in your head. We will ask you at the end of the block how many different series you have counted.” Subjects were stimulated with each of the four different block types according to a fixed order (AAAAA, AAAAB, BBBBB, BBBBA). Local effect ERP correlates are obtained by comparing local deviant trials to local standard ones across all blocks. Similarly, global effect contrasts global deviant trials to global standard trials across all blocks.

Supplementary Tables

ITEMS FOR MCS-	ITEMS FOR MCS+	ITEMS FOR EMCS
VISUAL FUNCTION SCALE<br>- Visual Pursuit<br>- Fixation<br><br>MOTOR FUNCTION SCALE<br>- Automatic Motor Response<br>- Object Manipulation<br>- Localization to Noxious Stimulation	AUDITORY FUNCTION SCALE<br>- Consistent Movement to Command<br>- Reproducible Movement to Command<br><br>VISUAL FUNCTION SCALE<br>- Object Recognition<br>- Object Localization: Reaching<br><br>OROMOTOR/VERBAL FUNCTION SCALE<br>- Intelligible Verbalization<br><br>COMMUNICATION SCALE<br>- Non-Functional: Intentional	MOTOR FUNCTION SCALE<br>- Functional Object Use<br><br>COMMUNICATION SCALE<br>- Functional: Accurate

Table S1: Details of the structured outcome phone interview: We questioned the corresponding contacts about the following items derived from the CRS-R. An item was considered as present only when the corresponding behavior was univocal, stable and obvious to detect.

	Global effect	Local Effect	Clinical status
**Se**	0.29 [0.21,0.37]	0.71 [0.62,0.78]	**0.89 [0.82,0.94]**
**Sp**	**0.88 [0.78,0.94]**	0.57 [0.45,0.69]	0.57 [0.45,0.69]
**VPP**	**0.81 [0.67,0.91]**	0.75 [0.66,0.82]	0.79 [0.71,0.85]
**VPN**	0.41 [0.33,0.49]	0.52 [0.41,0.64]	**0.74 [0.61,0.85]**
**LR+**	**2.38 [1.22,4.65]**	1.66 [1.25,2.20]	2.08 [1.59,2.72]
**LR-**	0.81 [0.71,0.93]	0.51 [0.37,0.71]	**0.20 [0.12,0.33]**
**BF**	19.06	810	$2.22*10^{10}$

Table S2: Performance of GE, LE and clinical status on consciousness recovery in all recordings of survivors only.

Numbers indicated in brackets correspond to the 95% confidence interval (CI).

	Global effect	Local Effect	Clinical status
**Se**	0.29 [0.21, 0.37]	0.71 [0.62, 0.78]	**0.89 [0.82, 0.94]**
**Sp**	**0.80 [0.73, 0.85]**	0.50 [0.43, 0.58]	0.63 [0.55, 0.70]
**VPP**	0.51 [0.39, 0.62]	0.51 [0.43, 0.58]	**0.63 [0.56, 0.70]**
**VPN**	0.61 [0.54, 0.67]	0.70 [0.62, 0.78]	**0.88 [0.82, 0.93]**
**LR+**	1.09 [0.90, 1.32]	1.42 [1.18, 1.71]	**2.39 [1.96, 2.91]**
**LR-**	0.90 [0.79, 1.02]	0.58 [0.43, 0.79]	**0.18 [0.11, 0.29]**
**BF**	1.35	314.84	$2.65*10^{19}$

Table S3: Performance of GE, LE and clinical status on consciousness recovery in all recordings (including deaths).

Numbers indicated in brackets correspond to the 95% confidence interval (CI).

	**Conscious**	**Unconscious**	**Total:**
**GE+**	32	8	40
**GE-**	60	43	103
**Total:**	92	51	143

Table S4: Table of contingency between GE and consciousness recovery in survivors.

	Global effect	Local Effect	Clinical status
**Se**	0.35 [0.25, 0.45]	0.68 [0.58, 0.78]	**0.89 [0.81, 0.95]**
**Sp**	**0.76** [0.69, 0.83]	0.51 [0.43, 0.60]	0.64 [0.55, 0.72]
**VPP**	0.48 [0.36, 0.61]	0.47 [0.39, 0.56]	**0.61 [0.52, 0.69]**
**VPN**	0.65 [0.57, 0.72]	0.72 [0.62, 0.80]	**0.90 [0.83, 0.95]**
**LR+**	1.47 [0.98, 2.21]	1.41 [1.13, 1.75]	**2.47 [1.96, 3.10]**
**LR-**	0.85 [0.72, 1.02]	0.61 [0.44, 0.86]	**0.17 [0.09, 0.31]**
**BF**	1.89	14.6	$5,6*10^{14}$

Table S5: Performance of GE, LE and clinical status on consciousness recovery in all patients (including deaths).

Numbers indicated in brackets correspond to the 95% confidence interval (CI).

Table S6: Summary table including all the patients with age, gender, etiology of brain lesion, delay between brain lesion and the evaluation, outcomes and the presence or not of local/global effect (view summary_table.csv)
